# 2-Carboxy­pyridinium hydrogen chloranilate

**DOI:** 10.1107/S1600536809006412

**Published:** 2009-02-28

**Authors:** Kazuma Gotoh, Hirokazu Nagoshi, Hiroyuki Ishida

**Affiliations:** a Department of Chemistry, Faculty of Science, Okayama University, Okayama 700-8530, Japan

## Abstract

In the crystal structure of the title salt, C_6_H_6_NO_2_^+^·C_6_HCl_2_O_4_^−^, the pyridine ring and the mean plane of the hydrogen chloranilate anion form a dihedral angle of 77.40 (8)°. The ionic components are held together by N—H⋯O and O—H⋯O hydrogen bonds, forming a supra­molecular ladder. C—H⋯O inter­actions are also present.

## Related literature

For the structures of related carboxy­pyridinium hydrogen chloranilates, see: Gotoh *et al.* (2006 ▶); Tabuchi *et al.* (2005 ▶); Ishida (2009 ▶).
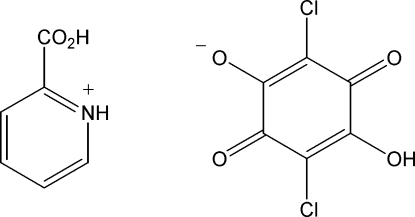


## Experimental

### 

#### Crystal data

C_6_H_6_NO_2_^+^·C_6_HCl_2_O_4_^−^
*M* *_r_* = 332.10
Monoclinic, 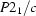

*a* = 9.4166 (8) Å
*b* = 19.6900 (16) Å
*c* = 6.7089 (6) Å
β = 99.043 (3)°
*V* = 1228.45 (18) Å^3^
*Z* = 4
Mo *K*α radiation
μ = 0.56 mm^−1^
*T* = 103 K
0.30 × 0.30 × 0.23 mm

#### Data collection

Rigaku R-AXIS RAPID-II diffractometer
Absorption correction: multi-scan (**ABSCOR**; Higashi, 1995 ▶) *T*_min_ = 0.847, *T*_max_ = 0.880
9710 measured reflections
3433 independent reflections
2228 reflections with *I* > 2σ(*I*)
*R*_int_ = 0.047

#### Refinement

*R*[*F* ^2^ > 2σ(*F* ^2^)] = 0.045
*wR*(*F* ^2^) = 0.144
*S* = 1.10
3433 reflections
202 parameters
H atoms treated by a mixture of independent and constrained refinement
Δρ_max_ = 0.47 e Å^−3^
Δρ_min_ = −0.92 e Å^−3^

### 

Data collection: *PROCESS-AUTO* (Rigaku/MSC, 2004 ▶); cell refinement: *PROCESS-AUTO*; data reduction: *CrystalStructure* (Rigaku/MSC, 2004 ▶); program(s) used to solve structure: *SHELXS97* (Sheldrick, 2008 ▶); program(s) used to refine structure: *SHELXL97* (Sheldrick, 2008 ▶); molecular graphics: *ORTEP-3* (Farrugia, 1997 ▶); software used to prepare material for publication: *CrystalStructure* and *PLATON* (Spek, 2009 ▶).

## Supplementary Material

Crystal structure: contains datablocks global, I. DOI: 10.1107/S1600536809006412/tk2376sup1.cif

Structure factors: contains datablocks I. DOI: 10.1107/S1600536809006412/tk2376Isup2.hkl

Additional supplementary materials:  crystallographic information; 3D view; checkCIF report

## Figures and Tables

**Table 1:** Hydrogen-bond geometry (Å, °)

*D*—H⋯*A*	*D*—H	H⋯*A*	*D*⋯*A*	*D*—H⋯*A*
N1—H1⋯O1	0.92 (4)	2.11 (3)	2.932 (2)	147 (3)
O2—H2⋯O5^i^	0.79 (3)	2.05 (3)	2.746 (2)	148 (3)
O6—H6⋯O4^ii^	0.90 (3)	1.63 (3)	2.528 (2)	177.1 (15)
C8—H8⋯O4^iii^	0.95	2.50	3.338 (3)	147
C9—H9⋯O3^iv^	0.95	2.33	3.227 (3)	156
C11—H11⋯O1^v^	0.95	2.46	3.374 (3)	162

Symmetry codes: (i) 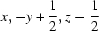; (ii) 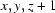; (iii) 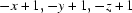; (iv) 
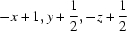
; (v) 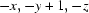.

**Fig. 1.**
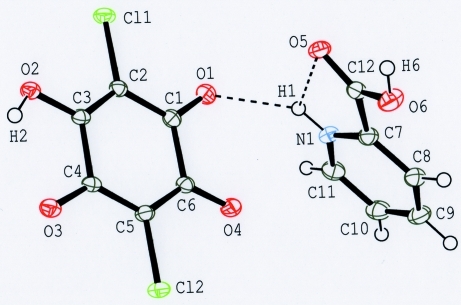
Molecular components of (I) showing the atom numbering scheme. Displacement ellipsoids are drawn at the 50% probability level. Intra- and inter-molecular N—H···O hydrogen bonds are indicated by dashed lines.

**Fig. 2.**
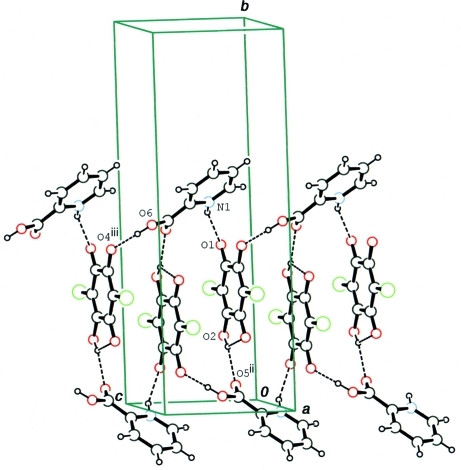
A partial packing diagram, viewed approximately along the a axis, showing the hydrogen-bonded supramolecular ladder. Dashed lines show N—H···O and O—H···O hydrogen bonds (symmetry codes as given in Table 1).

## supplementary crystallographic information

### Comment

The title salt, (I), was prepared in order to extend our study on D—H···A hydrogen bonding (D = N, O or C; A = N, O or Cl) in chloranilic acid – substituted-pyridine systems (Gotoh *et al.*, 2006; Tabuchi *et al.*, 2005).

Compound (I) comprises 2-carboxypyridinium cations and hydrogen chloranilate anions in the ratio 1:1. Ions directly connected by an N—H···O hydrogen bond, Fig. 1, form a dihedral angle between their respective mean planes of 77.40 (8)°. In the cation, the carboxy O5/O6/C12 plane forms a dihedral angle of 11.44 (6)° with the pyridine ring, which is similar to those of 2.74 (6) and 10.01 (3)° observed in 3-carboxypyridinium hydrogen chloranilate and 4-carboxypyridinium hydrogen chloranilate monohydrate, respectively (Ishida, 2009). The ions are further connected by O—H···O hydrogen bonds (Table 1) to afford a supramolecular ladder running along the *c* axis (Fig. 2). The ladders are linked by weaker N—H···O hydrogen bonds and C—H···O contacts to form a 3-D network (Table 1).

### Experimental

Crystals were obtained by slow evaporation from a methanol solution (*ca* 30 ml) containing a 1:1 molar ratio of chloranilic acid (0.302 g) and picolinic acid (0.179 g).

### Refinement

The H atoms attached to O and N were located from a difference Fourier map and refined isotropically to O—H = 0.79 (3) & 0.90 (3) Å and N—H = 0.92 (4) Å. The remaining H atoms were included in the riding approximation with C—H = 0.95 Å, and with *U*_iso_(H) = 1.2*U*_eq_(C).

### Crystal data

**Table:**

C_6_H_6_NO_2_^+^·C_6_HCl_2_O_4_^−^	*F*(000) = 672.00
*M**_r_* = 332.10	*D*_x_ = 1.795 Mg m^−^^3^
Monoclinic, *P*2_1_/*c*	Mo *K*α radiation, λ = 0.71075 Å
Hall symbol: -P 2ybc	Cell parameters from 7392 reflections
*a* = 9.4166 (8) Å	θ = 3.0–30.0°
*b* = 19.6900 (16) Å	µ = 0.56 mm^−^^1^
*c* = 6.7089 (6) Å	*T* = 103 K
β = 99.043 (3)°	Platelet, dark purple
*V* = 1228.45 (18) Å^3^	0.30 × 0.30 × 0.23 mm
*Z* = 4	

### Data collection

**Table:**

Rigaku R-AXIS RAPID-II diffractometer	2228 reflections with *I* > 2σ(*I*)
Detector resolution: 10.00 pixels mm^-1^	*R*_int_ = 0.047
ω scans	θ_max_ = 30.0°
Absorption correction: multi-scan (*ABSCOR*; Higashi, 1995)	*h* = −13→13
*T*_min_ = 0.847, *T*_max_ = 0.880	*k* = −27→27
9710 measured reflections	*l* = −8→9
3433 independent reflections	

### Refinement

**Table:**

Refinement on *F*^2^	Secondary atom site location: difference Fourier map
*R*[*F*^2^ > 2σ(*F*^2^)] = 0.045	Hydrogen site location: inferred from neighbouring sites
*wR*(*F*^2^) = 0.144	H atoms treated by a mixture of independent and constrained refinement
*S* = 1.10	*w* = 1/[σ^2^(*F*_o_^2^) + (0.0659*P*)^2^ + 0.8975*P*] where *P* = (*F*_o_^2^ + 2*F*_c_^2^)/3
3433 reflections	(Δ/σ)_max_ < 0.001
202 parameters	Δρ_max_ = 0.47 e Å^−^^3^
0 restraints	Δρ_min_ = −0.92 e Å^−^^3^
Primary atom site location: structure-invariant direct methods	

### Special details

**Table:**

Geometry. All e.s.d.'s (except the e.s.d. in the dihedral angle between two l.s. planes) are estimated using the full covariance matrix. The cell e.s.d.'s are taken into account individually in the estimation of e.s.d.'s in distances, angles and torsion angles; correlations between e.s.d.'s in cell parameters are only used when they are defined by crystal symmetry. An approximate (isotropic) treatment of cell e.s.d.'s is used for estimating e.s.d.'s involving l.s. planes.
Refinement. Refinement of *F*^2^ against ALL reflections. The weighted *R*-factor *wR* and goodness of fit *S* are based on *F*^2^, conventional *R*-factors *R* are based on *F*, with *F* set to zero for negative *F*^2^. The threshold expression of *F*^2^ > σ(*F*^2^) is used only for calculating *R*-factors(gt) *etc*. and is not relevant to the choice of reflections for refinement. *R*-factors based on *F*^2^ are statistically about twice as large as those based on *F*, and *R*- factors based on ALL data will be even larger.

### Fractional atomic coordinates and isotropic or equivalent isotropic displacement parameters (Å^2^)

**Table:**

	*x*	*y*	*z*	*U*_iso_*/*U*_eq_	
Cl1	−0.17894 (6)	0.28996 (3)	0.27912 (9)	0.02060 (16)	
Cl2	0.46528 (6)	0.29140 (3)	0.10812 (9)	0.02077 (16)	
O1	0.00723 (19)	0.40867 (8)	0.2367 (3)	0.0218 (4)	
O2	0.01147 (19)	0.17042 (9)	0.2397 (3)	0.0184 (3)	
O3	0.27117 (18)	0.17271 (8)	0.1488 (3)	0.0196 (4)	
O4	0.27772 (19)	0.41277 (8)	0.1577 (3)	0.0209 (4)	
O5	0.12383 (18)	0.45615 (9)	0.6917 (3)	0.0215 (4)	
O6	0.3466 (2)	0.47658 (10)	0.8601 (3)	0.0265 (4)	
N1	0.1860 (2)	0.52690 (11)	0.3720 (3)	0.0199 (4)	
C1	0.0667 (2)	0.35416 (11)	0.2174 (3)	0.0164 (4)	
C2	−0.0036 (2)	0.28950 (11)	0.2347 (3)	0.0160 (4)	
C3	0.0678 (2)	0.23079 (11)	0.2188 (3)	0.0154 (4)	
C4	0.2206 (2)	0.22965 (11)	0.1715 (3)	0.0155 (4)	
C5	0.2899 (2)	0.29272 (11)	0.1544 (4)	0.0170 (4)	
C6	0.2232 (2)	0.35480 (12)	0.1732 (3)	0.0165 (4)	
C7	0.2794 (3)	0.52837 (12)	0.5464 (4)	0.0198 (5)	
C8	0.3984 (3)	0.56970 (13)	0.5626 (4)	0.0229 (5)	
H8	0.4656	0.5708	0.6840	0.027*	
C9	0.4194 (3)	0.60996 (13)	0.3987 (4)	0.0262 (5)	
H9	0.5010	0.6388	0.4077	0.031*	
C10	0.3206 (3)	0.60759 (13)	0.2230 (4)	0.0268 (5)	
H10	0.3332	0.6352	0.1109	0.032*	
C11	0.2035 (3)	0.56488 (13)	0.2118 (4)	0.0239 (5)	
H11	0.1357	0.5624	0.0913	0.029*	
C12	0.2418 (3)	0.48289 (12)	0.7090 (4)	0.0195 (5)	
H1	0.109 (5)	0.498 (2)	0.363 (6)	0.053 (11)*	
H2	0.071 (4)	0.1437 (17)	0.227 (5)	0.032 (9)*	
H6	0.319 (4)	0.4534 (18)	0.964 (6)	0.043 (10)*	

### Atomic displacement parameters (Å^2^)

**Table:**

	*U*^11^	*U*^22^	*U*^33^	*U*^12^	*U*^13^	*U*^23^
Cl1	0.0146 (3)	0.0253 (3)	0.0233 (3)	0.0013 (2)	0.0076 (2)	−0.0006 (2)
Cl2	0.0151 (3)	0.0258 (3)	0.0230 (3)	−0.0018 (2)	0.0080 (2)	−0.0011 (2)
O1	0.0207 (8)	0.0196 (8)	0.0256 (9)	0.0020 (7)	0.0051 (7)	−0.0022 (7)
O2	0.0163 (8)	0.0164 (7)	0.0238 (9)	0.0013 (7)	0.0074 (7)	−0.0008 (7)
O3	0.0176 (8)	0.0201 (8)	0.0220 (8)	0.0016 (7)	0.0063 (7)	−0.0008 (7)
O4	0.0232 (9)	0.0200 (8)	0.0210 (9)	−0.0027 (7)	0.0083 (7)	0.0019 (6)
O5	0.0177 (8)	0.0212 (8)	0.0274 (9)	−0.0017 (7)	0.0094 (7)	0.0018 (7)
O6	0.0221 (9)	0.0336 (10)	0.0235 (9)	−0.0063 (8)	0.0024 (7)	0.0050 (8)
N1	0.0145 (9)	0.0229 (10)	0.0229 (10)	−0.0018 (8)	0.0050 (8)	0.0000 (8)
C1	0.0182 (10)	0.0180 (10)	0.0130 (10)	0.0007 (9)	0.0020 (8)	0.0001 (8)
C2	0.0130 (10)	0.0197 (10)	0.0152 (10)	0.0009 (8)	0.0023 (8)	−0.0019 (8)
C3	0.0154 (10)	0.0183 (10)	0.0132 (10)	−0.0002 (8)	0.0048 (8)	0.0005 (8)
C4	0.0149 (10)	0.0193 (10)	0.0132 (10)	0.0009 (8)	0.0048 (8)	0.0003 (8)
C5	0.0132 (10)	0.0207 (10)	0.0176 (10)	−0.0013 (9)	0.0041 (8)	−0.0006 (9)
C6	0.0165 (10)	0.0204 (10)	0.0133 (10)	−0.0014 (9)	0.0039 (8)	−0.0011 (8)
C7	0.0179 (11)	0.0184 (11)	0.0241 (12)	0.0001 (9)	0.0067 (9)	−0.0006 (9)
C8	0.0188 (11)	0.0236 (12)	0.0272 (13)	0.0000 (10)	0.0066 (10)	−0.0018 (10)
C9	0.0223 (12)	0.0231 (12)	0.0355 (14)	−0.0031 (10)	0.0118 (11)	0.0023 (11)
C10	0.0292 (13)	0.0248 (12)	0.0291 (13)	0.0006 (11)	0.0129 (11)	0.0049 (10)
C11	0.0229 (12)	0.0270 (12)	0.0221 (12)	0.0049 (10)	0.0051 (10)	0.0028 (10)
C12	0.0210 (11)	0.0193 (10)	0.0197 (11)	−0.0002 (9)	0.0076 (9)	−0.0021 (9)

### Geometric parameters (Å, °)

**Table:**

Cl1—C2	1.723 (2)	C1—C6	1.548 (3)
Cl2—C5	1.727 (2)	C2—C3	1.350 (3)
O1—C1	1.227 (3)	C3—C4	1.521 (3)
O2—C3	1.318 (3)	C4—C5	1.416 (3)
O2—H2	0.79 (3)	C5—C6	1.389 (3)
O3—C4	1.237 (3)	C7—C8	1.375 (3)
O4—C6	1.263 (3)	C7—C12	1.497 (3)
O5—C12	1.218 (3)	C8—C9	1.394 (4)
O6—C12	1.305 (3)	C8—H8	0.9500
O6—H6	0.90 (4)	C9—C10	1.383 (4)
N1—C11	1.340 (3)	C9—H9	0.9500
N1—C7	1.349 (3)	C10—C11	1.379 (4)
N1—H1	0.92 (4)	C10—H10	0.9500
C1—C2	1.448 (3)	C11—H11	0.9500
			
C3—O2—H2	107 (3)	O4—C6—C1	115.8 (2)
C12—O6—H6	112 (2)	C5—C6—C1	117.90 (19)
C11—N1—C7	122.5 (2)	N1—C7—C8	119.6 (2)
C11—N1—H1	119 (2)	N1—C7—C12	115.0 (2)
C7—N1—H1	118 (2)	C8—C7—C12	125.4 (2)
O1—C1—C2	122.6 (2)	C7—C8—C9	119.3 (2)
O1—C1—C6	118.5 (2)	C7—C8—H8	120.4
C2—C1—C6	118.91 (19)	C9—C8—H8	120.4
C3—C2—C1	120.4 (2)	C10—C9—C8	119.5 (2)
C3—C2—Cl1	121.40 (18)	C10—C9—H9	120.2
C1—C2—Cl1	118.15 (17)	C8—C9—H9	120.2
O2—C3—C2	123.3 (2)	C11—C10—C9	119.5 (2)
O2—C3—C4	114.75 (19)	C11—C10—H10	120.3
C2—C3—C4	121.9 (2)	C9—C10—H10	120.3
O3—C4—C5	126.4 (2)	N1—C11—C10	119.7 (2)
O3—C4—C3	115.7 (2)	N1—C11—H11	120.2
C5—C4—C3	117.85 (19)	C10—C11—H11	120.2
C6—C5—C4	122.9 (2)	O5—C12—O6	126.9 (2)
C6—C5—Cl2	119.23 (17)	O5—C12—C7	120.4 (2)
C4—C5—Cl2	117.86 (17)	O6—C12—C7	112.7 (2)
O4—C6—C5	126.3 (2)		
			
O1—C1—C2—C3	−177.9 (2)	C4—C5—C6—C1	0.7 (3)
C6—C1—C2—C3	2.1 (3)	Cl2—C5—C6—C1	−179.21 (16)
O1—C1—C2—Cl1	1.3 (3)	O1—C1—C6—O4	−0.9 (3)
C6—C1—C2—Cl1	−178.77 (16)	C2—C1—C6—O4	179.2 (2)
C1—C2—C3—O2	177.7 (2)	O1—C1—C6—C5	179.2 (2)
Cl1—C2—C3—O2	−1.4 (3)	C2—C1—C6—C5	−0.7 (3)
C1—C2—C3—C4	−3.3 (3)	C11—N1—C7—C8	−0.6 (4)
Cl1—C2—C3—C4	177.58 (16)	C11—N1—C7—C12	179.6 (2)
O2—C3—C4—O3	3.3 (3)	N1—C7—C8—C9	0.7 (4)
C2—C3—C4—O3	−175.8 (2)	C12—C7—C8—C9	−179.5 (2)
O2—C3—C4—C5	−177.73 (19)	C7—C8—C9—C10	−0.1 (4)
C2—C3—C4—C5	3.2 (3)	C8—C9—C10—C11	−0.7 (4)
O3—C4—C5—C6	177.0 (2)	C7—N1—C11—C10	−0.2 (4)
C3—C4—C5—C6	−1.8 (3)	C9—C10—C11—N1	0.8 (4)
O3—C4—C5—Cl2	−3.0 (3)	N1—C7—C12—O5	−11.4 (3)
C3—C4—C5—Cl2	178.11 (16)	C8—C7—C12—O5	168.8 (2)
C4—C5—C6—O4	−179.2 (2)	N1—C7—C12—O6	168.4 (2)
Cl2—C5—C6—O4	0.8 (3)	C8—C7—C12—O6	−11.4 (3)

### Hydrogen-bond geometry (Å, °)

**Table:**

*D*—H···*A*	*D*—H	H···*A*	*D*···*A*	*D*—H···*A*
N1—H1···O1	0.92 (4)	2.11 (3)	2.932 (2)	147 (3)
N1—H1···O5	0.92 (4)	2.33 (4)	2.698 (2)	103 (2)
N1—H1···O5^i^	0.92 (4)	2.34 (4)	2.900 (2)	119 (3)
O2—H2···O3	0.79 (3)	2.11 (3)	2.612 (2)	122 (3)
O2—H2···O5^ii^	0.79 (3)	2.05 (3)	2.746 (2)	148 (3)
O6—H6···O4^iii^	0.90 (3)	1.63 (3)	2.528 (2)	177.1 (15)
C8—H8···O4^iv^	0.95	2.50	3.338 (3)	147
C9—H9···O3^v^	0.95	2.33	3.227 (3)	156
C11—H11···O1^vi^	0.95	2.46	3.374 (3)	162

Symmetry codes: (i) −*x*, −*y*+1, −*z*+1; (ii) *x*, −*y*+1/2, *z*−1/2; (iii) *x*, *y*, *z*+1; (iv) −*x*+1, −*y*+1, −*z*+1; (v) −*x*+1, *y*+1/2, −*z*+1/2; (vi) −*x*, −*y*+1, −*z*.
